# Do timing and frequency of antenatal care make a difference in maternal micronutrient intake and breastfeeding practices? Insights from a multi-country study in South Asia

**DOI:** 10.1371/journal.pgph.0002993

**Published:** 2024-03-04

**Authors:** Md Jahirul Islam, Khondker Mohammad Zobair

**Affiliations:** 1 Griffith Criminology Institute, Griffith University, Brisbane, Queensland, Australia; 2 Ministry of Public Administration, Bangladesh Secretariat, Dhaka, Bangladesh; 3 Department of International Business and Asian Studies, Griffith University, Brisbane, Queensland, Australia; Yale University, UNITED STATES

## Abstract

Despite the established benefits of vitamins and minerals for maternal and neonatal health, global micronutrient deficiency remains a significant concern. As such, the World Health Organization advocates timely antenatal care (ANC) initiation and micronutrient supplementation for expectant mothers. This study investigates the association between ANC timing and frequency and maternal health behaviours, specifically iron-folic acid (IFA) intake, early breastfeeding initiation, and exclusive breastfeeding among married women in South Asia. By utilizing recent Demographic and Health Survey data, this study focuses on married women aged 15–49 in Bangladesh (*N* = 966), India (*N* = 89,472), and Pakistan (*N* = 1,005), specifically primiparous women with children aged 0–23 months living with the motherMultivariable analysis revealed that women receiving ≥4 ANC visits were more likely to consume IFA ≥90 days compared to those with fewer visits in Bangladesh (AOR: 1.85, 95% CI [1.30, 2.63]), India (AOR: 1.87, 95% CI [1.81, 1.94]), and Pakistan (AOR: 1.92, 95% CI [1.24, 2.97]). Women receiving first ANC in the second or third trimester were less likely to consume IFC for ≥90 days compared to those with first-trimester ANC. While the ANC timing did not significantly influence early breastfeeding initiation, ANC frequency was inversely associated with delayed initiation in all countries. Breastfeeding advice during ANC visits was significantly associated with reduced odds of delayed breastfeeding initiation. Neither ANC timing nor frequency significantly predicted exclusive breastfeeding, except for breastfeeding advice in India. This study highlights the importance of ANC in maternal and child health outcomes. ANC timing and frequency, along with breastfeeding advice during ANC, notably influence maternal IFA consumption and early breastfeeding initiation. These findings underscore the need for targeted interventions during ANC visits to enhance maternal and child health practices in low- and middle-income countries.

## Introduction

Micronutrient deficiency (MND) poses a significant global health concern, particularly among women of reproductive age in low- and middle-income countries [1–3]. Micronutrients—which include several vitamins and minerals, such as iron, zinc, vitamin A, iodine, and folate—are essential for normal metabolism, growth, and physical well-being [4]. MNDs during pregnancy are associated with pregnancy complications and increased risk of fatal and maternal morbidity and mortality [5,6]. The “first 1000 days”, spanning from a woman’s pregnancy to her child’s second birthday, offer a critical window of opportunity to ensure optimal health and nutrition for the child [7]. Notably, the nutritional status of a woman during pregnancy and postpartum is a key indicator of her child’s subsequent health outcomes, with poor nutrition linked to the intergenerational transmission of ill health [8–10].

South Asia stands as a hotspot for the global burden of malnutrition and micronutrient deficiencies among women and children [11]. Alarmingly, 38% (*n* = 32 million) of global pregnant women are anaemic, with the second-highest prevalence (52%) in South Asia [3,11]. This region is home to the highest number of stunted and overweight children under five [12]. Moreover, 58% of newborns are not breastfed immediately after birth and 41% of infants are not exclusively breastfed [13]. Evidence suggests that maternal multiple micronutrient supplementation during pregnancy, including Iron and Folic Acid (IFA), can save approximately 102,000 lives per year globally [7]. The inclusion of IFA in maternal supplementation contributes significantly to reducing maternal anaemia and preventing neural tube defects in the developing featus, highlighting its critical impact on maternal and child health [5–7]. Because the provision of food supplements is not logistically and economically viable in resource-constrained settings [1], the World Health Organization (WHO) recommends multiple micronutrient supplements for pregnant women and breastfeeding for up to 2 years as an alternative [14].

Given the significant contribution of micronutrient consumption and breastfeeding for improving maternal and child health, research has begun to identify their determinants, particularly those that are potentially modifiable through health policy interventions. Antenatal care (ANC) emerges as a notable factor, offering a platform for counselling on maternal and neonatal health and nutrition [15]. ANC also provides a great opportunity to mentally prepare the mother to initiate breastfeeding within 1h after childbirth, exclusively breastfeed for 6 months and continue breastfeeding for an additional 18 months or longer with complementary foods [16]. Recently, the WHO recommends that pregnant women should receive the first ANC visit within 12 weeks of conception and an additional seven visits throughout the gestational period because early initiation of the first ANC offers health workers the opportunity to provide timely information and services as per the gestational age and health condition. Previous research has revealed an association between the number of ANC visits or timing of the first ANC initiation and pregnancy outcomes, such as preterm birth, stillbirth, and low birthweight [17–20].

Earlier studies on the impact of ANC have primarily been carried out in high-income countries, focusing predominantly on birth outcomes [17]. Notably, these findings were inconclusive, reporting either modest [17] or no effects [18] on birth outcomes. The variability in methodologies, tools, and sample populations across these studies has posed challenges in drawing meaningful comparisons. Yet there has been a noticeable scarcity of research exploring the influence of ANC on maternal and child nutritional status in low- and middle-income settings, especially in the dynamic context of South Asia. A recent research [21] in Uttar Pradesh, India, addressed this gap by examining various maternal and child health outcomes related to ANC, yet a comprehensive understanding, especially using nationally representative data, remains elusive in this region. To bridge these research gaps, this study utilizes nationally representative data to investigate the influence of ANC timing and number of visits on maternal micronutrient intake and breastfeeding practices (early initiation, and exclusive breastfeeding) among currently married women in three South Asian countries: Bangladesh, India, and Pakistan. Examining this association is crucial to breaking the cycle of intergenerational transmission of malnutrition from mothers to offspring.

## Methods

### Data sources

This study is a secondary analysis of the most recent waves of Demographic and Health Surveys (DHS) in Bangladesh (2017–2018), India (2015–2016), and Pakistan (2017–2018). DHS data are nationally representative, household surveys of ever-married women aged 15–49 years. Using a standardized questionnaire, this survey covers a variety of demographic, health, and well-being topics in low- and middle-income countries. A two-stage cluster sampling method is applied to select urban and rural households. The detailed methodology regarding each of the surveys is available elsewhere [22–24]. Analyses were limited to only primiparous women who had a child of 0–23 months old and currently living with the mother. For this analysis, we dropped cases with missing data for the variables included in this study, which left data for 966, 89,472, and 1005 participants for Bangladesh, India, and Pakistan, respectively as the analytical sample.

### Outcome variables

#### Consumption of iron and folic acid (IFA)

To provide an assessment of micronutrient supplementation intake, we analyzed the reception of the total number of iron and folic acid (IFA) tablets as outcome variables. Women were asked, “During the last pregnancy, for how many days did you take the tablets or syrup?” The consumption of IFA was measured with a dichotomous variable coded as follows: < 90 tablets = 0 versus ≥ 90 tablets = 1.

It should be noted that while the WHO recommends at least 180 IFA supplements beginning in the first trimester of pregnancy, many countries opt for 90 or more [25]. The decision to use ‘90 IFA’ tablets as the cutoff was based on alignment with common practices in maternal health research [26–28]. However, we acknowledge the importance of considering international guidelines for IFA supplementation and our chosen cutoff is not intended as a one-size-fits-all standard but rather as a pragmatic approach for analytical purposes.

#### Early initiation of breastfeeding

Early initiation of breastfeeding was determined by asking whether newborns were breastfed within the first hour of birth, and coded as 0 "if the mother initiated breast milk within the first hour of birth" and 1 "otherwise".

#### Exclusive breastfeeding (EBF)

To define EBF, the WHO’s definition was used: “up to six months of age, the infant receives only breast milk without any additional liquids or solids—not even water—except oral rehydration solution, or drops/syrups of vitamins, minerals, or medicines” [29, p.2]. EBF was determined based on responses to questions that assessed whether the woman had ever breastfed her infant, whether she was currently breastfeeding, and if so, whether any other solid or liquid was fed to the infant during the last 24 hours at the time of survey. The feeding practice was coded as either EBF (= 1) if a baby had received only breastmilk with no other additional food for up to six months, or non-EBF (= 0) if a child had received anything other than breastmilk.

### Exposure variables

#### Timing of first ANC visit

Respondents were asked about whether they saw anyone for ANC during the pregnancy preceding their most recent live birth, and if they did, how many months pregnant they were when they first received ANC. The timing of the women’s first ANC visit was categorized, based on WHO recommendations, as occurring in the first trimester of pregnancy (1–3 months) = 0, second trimester (4–6 months) = 1, and third trimester (≥4 months) = 2.

#### Number of ANC visits

We used the number of visits as a continuous variable and created categories based on the previous four-visit ANC model [30]. Women were asked how many times they received ANC during the pregnancy. The number of ANC visits was categorized based on the previous four-visit ANC model as <4 visits = 0 and ≥4 visits = 1.

#### Breastfeeding advice during ANC

Respondents were asked whether they received advice on breastfeeding at least once during any of the ANC visits in the last three months of the last pregnancy (no = 0 versus yes = 1).

### Control variables

A range of theoretically and empirically associated socio-demographic variables were included in this study [15,31]. Respondent’s age was categorized into three categories: adolescent (≤19 years = 0), young adults (20–24 years = 1), and adults (≥25 = 2). The women’s educational level was defined as: no education (= 0), primary (= 1), or secondary (= 2), and higher (= 3). The area of residence was categorized as urban (0) versus rural (1). Wealth was categorized as poor (individuals falling into the lowest and second wealth quintiles, 0), middle (those falling into the middle wealth quintile, 1), or rich (those falling into the fourth and highest wealth quintiles, 2) category. Pregnancy intendedness was coded as intended = 0 and unintended = 1. Mass media exposure (watching TV) was coded as not at all (respondents who reported no TV watching) = 0, irregular (who reported watching TV but less frequently than once a week) = 1, and regular (who reported watching TV at least once a week) = 2.

### Ethical approval

All procedures and questionnaires for standard DHS surveys underwent a comprehensive review and received endorsement from the ICF Institutional Review Board at Calverton in the USA. In addition, an Ethical Review Board granted approval for each survey conducted within the country. The respondents provided informed consent for individual interviews and all information was collected confidentially. This research was considered exempt from a full review because it relied on an anonymous public use of a secondary dataset that contained no identifiable information about the survey participants.

### Statistical analyses

All statistical analyses were conducted using IBM SPSS V24.0, with statistical significance set at *p* < 0.05 (2-tailed). Descriptive statistics for the predictive characteristics and outcome measures were estimated. The relationships between the predictive characteristics and outcome measures were assessed using cross-tabulations and the *χ*^2^ test. The multicollinearity of the variables was checked by examining the variance inflation factors (VIFs<2.5 but found no evidence of multicollinearity.

To estimate adjusted odds ratios (AOR) and 95% confidence intervals to compare the strength of the associations between the outcome and exposure variables, we designed three adjusted multivariable logistic regression models—one separate model for each of the outcome variables (maternal consumption of IFA, early initiation of breastfeeding, and exclusive breastfeeding) with the exposure variables (timing of first ANC, number of ANC visits, and breastfeeding advice during ANC). All the covariates were entered simultaneously into the multiple logistic regression models.

## Results

### Descriptive statistics

**Table 1** outlines a descriptive overview of women’s characteristics in Bangladesh, India, and Pakistan. Adult women accounted for 47.5–67.0% in all countries. One in two Pakistani women (44.6%) had no education, compared to one in four women (24.7%) in India. Wealth index distribution varied, with most Bangladeshi women falling into the rich category, while Indian and Pakistani women were predominantly in the poor category. Rural residency was common across countries, encompassing over half of the women. Last pregnancy intentions indicated that 6.8% to 15.5% of women experienced unintended pregnancies. TV viewership differed, with two in five Pakistani women never watching TV compared to one in four Indian women.

**Table 1:** Sample characteristics, by country: Bangladesh (*N* = 966), India (*N* = 89,472), and Pakistan (*N* = 1005).

Characteristics	Bangladesh		India		Pakistan	
	*n*	%	*n*	%	*n*	%
**Maternal age**						
15–19	214	22.2	3,550	4.0	119	11.8
20–24	293	30.3	26,012	29.1	279	27.8
25–49	459	47.5	59,910	67.0	607	60.4
**Education**						
No education	39	4.0	22,071	24.7	448	44.6
Primary education	192	19.9	11,437	12.8	117	11.6
Secondary education	492	50.9	44,306	49.5	241	24.0
Higher education	243	25.2	11,658	13.0	199	19.8
**Wealth index category**						
Poor	191	19.8	38,989	43.6	410	40.8
Middle	205	21.2	18,023	20.1	206	20.5
Rich	570	59.0	32,460	36.3	389	38.7
**Area of residence**						
Urban	469	48.6	25,045	28.0	484	48.2
Rural	497	51.4	64,427	72.0	521	51.8
**Pregnancy intendedness**						
Intended	816	84.5	83,385	93.2	937	93.2
Unintended	150	15.5	6,087	6.8	68	6.8
**Mass media exposure: Watching TV**						
Not at all	47	4.9	23,346	26.1	407	40.5
Irregularly	24	2.5	17,044	19.0	111	11.0
Regularly	895	92.7	49,082	54.9	487	48.5
**Number of ANC visits**						
<4	378	39.1	42,470	47.5	409	40.7
≥4	588	60.9	47,002	52.5	596	59.3
**Timing of first ANC visit**						
First Trimester	444	47.0	55,035	72.1	639	70.8
Second Trimester	365	38.7	17,818	23.4	217	24.0
Third Trimester	135	14.3	3,451	4.5	47	5.2
**Received breastfeeding advice during ANC visit**						
No	79	12.8	8,671	19.4	369	40.5
Yes	537	87.2	36,088	80.6	541	59.5

*ANC* Antenatal Care.

Receiving ANC varied across the studied countries. In India, 47.5% of women had less than four ANC visits, compared to 39.1% in Bangladesh and 40.7% in Pakistan. Over 70.0% of Indian and Pakistani women received the first ANC in the first trimester, compared to 47.0% in Bangladesh. Regarding receiving breastfeeding advice during ANC visits, the rate ranged from 59.5% in Pakistan to 87.2% in Bangladesh.

**Fig 1:**
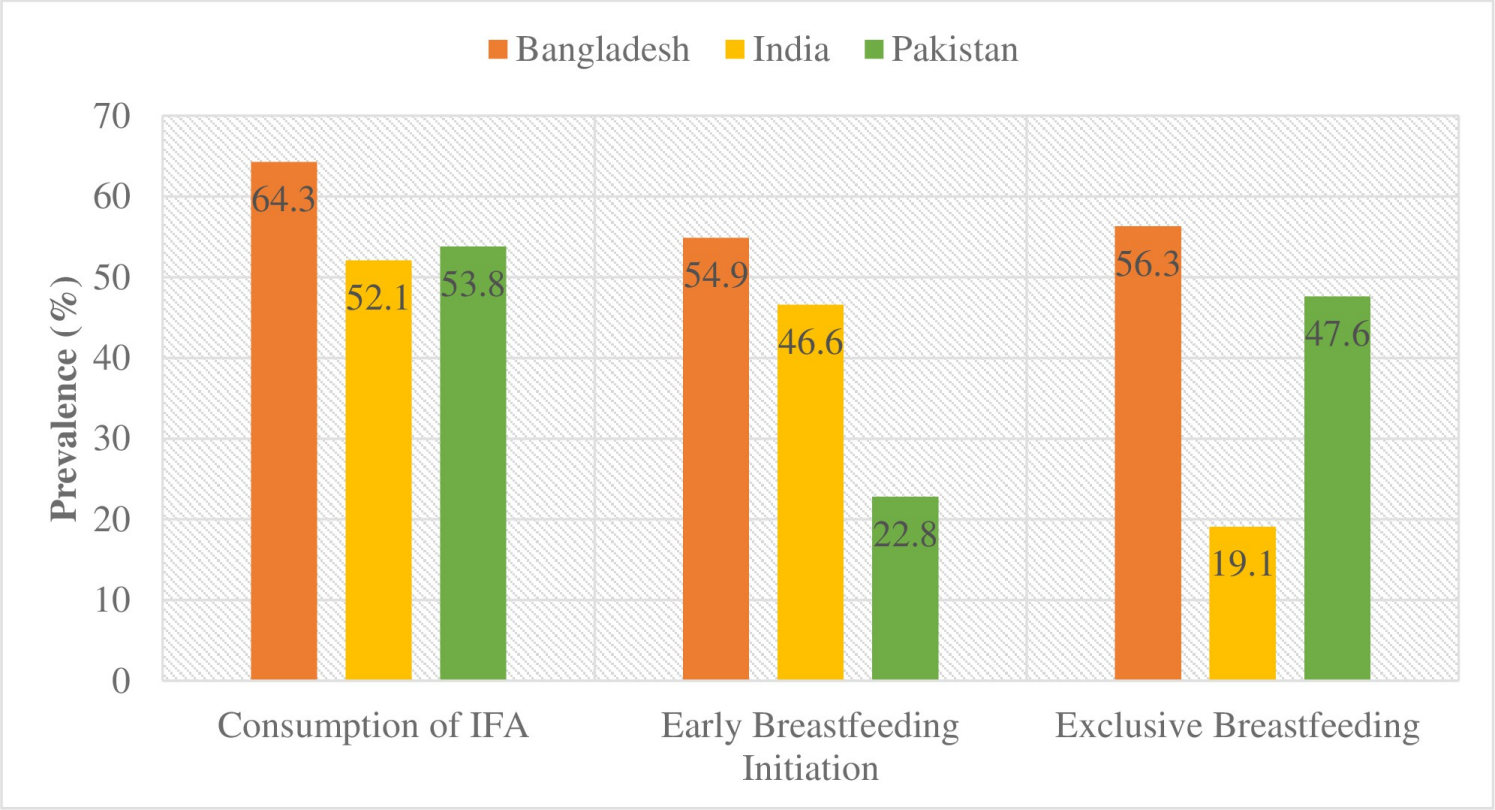
The prevalence of consumption of IFA, initiation of early breastfeeding, and exclusive breastfeeding by countries.

Fig 1 displays the prevalence of IFA consumption, early breastfeeding initiation, and exclusive breastfeeding across countries. IFA consumption varied from 52.1% in India to 64.3% in Bangladesh. Early initiation of breastfeeding was prominent in Bangladesh (54.9%) but limited in Pakistan (22.8%). Exclusive breastfeeding ranged from 19.1% in India to 56.3% in Bangladesh.

S1 Table demonstrates the socio-demographic differentials for the outcome variables by country. Notably, the associations between these characteristics and outcomes exhibited considerable disparities among the countries.

### Multivariable analyses

Table 2 shows the results of multivariable analyses exploring the association between ANC timing/frequency and women’s behaviour in terms of IFA consumption and breastfeeding practices. The adjusted multivariable logistic regression model reveals that women receiving four or more ANC visits were significantly more likely to consume IFA for ≥90 days compared to those with fewer visits in all countries: Bangladesh (AOR: 1.85, 95% CI [1.30, 2.63]), India (AOR: 1.87, 95% CI [1.81, 1.94]), and Pakistan (AOR: 1.92, 95% CI [1.24, 2.97]). Compared to first-trimester ANC, second or third-trimester ANC was associated with lower IFC consumption for ≥90 days across all countries.

**Table 2:** Adjusted odds ratios for associations between timing and number of ANC visits and micronutrient intake and breastfeeding performance among currently married women of Bangladesh, India, and Pakistan.

Country	Characteristics	Adjusted odds ratio (95% confidence interval)		
		Consumption of IFA^1^	Breastfeeding initiation^1^	Exclusive Breastfeeding^1^
**Bangladesh**	**Number of ANC visits**			
	<4 visits	1.00	1.00	1.00
	≥4 visits	1.85 (1.30–2.63) ^**b**^	0.58 (0.39–0.88) ^**b**^	1.06 (0.44–2.55)
	**Timing of 1**^**st**^ **ANC visit**			
	First Trimester	1.00	1.00	1.00
	Second Trimester	0.72 (0.51–1.02) ^**c**^	0.76 (0.53–1.11)	0.37 (0.16–0.82)
	Third Trimester	0.50 (0.30–0.84) ^**c**^	0.59 (0.32–1.10)	0.57 (0.14–2.27)
	**Received breastfeeding advice during ANC**			
	No	-	1.00	1.00
	Yes	-	0.72 (0.65–0.80) ^**b**^	0.45 (0.13–1.62)
**India**	**Number of ANC visits**			
	<4 visits	1.00	1.00	1.00
	≥4 visits	1.87 (1.81–1.94) ^**a**^	0.77 (0.74–0.80) ^**a**^	0.96 (0.88–1.04)
	**Timing of 1**^**st**^ **ANC visit**			
	First Trimester	1.00	1.00	1.00
	Second Trimester	0.80 (0.76–0.83) ^**a**^	1.09 (1.04–1.15)	0.94 (0.96–1.03)
	Third Trimester	0.88 (0.81–0.95) ^**b**^	1.06 (0.96–1.17)	1.20 (1.01–1.43)
	**Received breastfeeding advice during ANC**			
	No	-	1.00	1.00
	Yes	-	0.69 (0.66–0.73) ^**a**^	1.18 (1.06–1.30) ^**b**^
**Pakistan**	**Number of ANC visits**			
	<4 visits	1.00	1.00	1.00
	≥4 visits	1.92 (1.24–2.97) ^**c**^	0.89 (0.51–1.25) ^**b**^	1.18 (0.56–2.47)
	**Timing of 1**^**st**^ **ANC visit**			
	First Trimester	1.00	1.00	1.00
	Second Trimester	1.02 (0.65–1.61)	0.75 (0.48–1.17)	1.13 (0.50–2.53)
	Third Trimester	0.07 (0.01–0.53) ^**c**^	0.73 (0.31–1.70)	0.43 (0.11–1.70)
	**Received breastfeeding advice during ANC**			
	No	-	1.00	1.00
	Yes	-	0.58 (0.40–0.84) ^**c**^	0.89 (0.48–1.66)

^1^Adjusted for age, education, wealth, residence, pregnancy intendedness, and frequency of watching TV.

Here ^a^*p*< 0.001

^b^*p*< 0.01

^c^*p*< 0.05.

Regarding breastfeeding initiation, women with four or more ANC visits exhibited lower odds of delayed initiation across all countries [Bangladesh, (AOR: 0.58, 95% CI [0.39, 0.88]); India, (AOR: 0.77, 95% CI [0.74, 0.80]); and Pakistan, (AOR: 0.89, 95% CI [0.51, 1.25])]. The ANC timing did not significantly influence the early initiation of breastfeeding in all the countries. However, consistent across all countries, women who received advice on breastfeeding during ANC had a lower likelihood of delayed breastfeeding initiation compared to those who received no advice.

For exclusive breastfeeding, ANC timing and frequency showed no significant influence. Although not significant in Bangladesh and Pakistan, Indian women who received breastfeeding advice during ANC were 1.18 times more likely to exclusively breastfeed compared to those without such advice.

## Discussion

This research aimed to explore the influence of the timing and frequency of antenatal care on maternal micronutrient intake and breastfeeding practices in a nationally representative sample of married women in Bangladesh, India, and Pakistan. The study unveiled a nuanced link between ANC utilization and maternal health behaviours, with significant cross-country variations. Additionally, our analyses show that only the number of ANC visits and breastfeeding advice during ANC were positively associated with early initiation of breastfeeding. Breastfeeding advice during ANC was positively connected with exclusive breastfeeding only in India. Overall, this study adds to a limited but growing body of evidence on the impacts of ANC on maternal and child nutrition in developing countries. This association suggests the potential health benefits of adhering to recommended ANC timing and numbers, highlighting improvements in maternal anaemia, infant nutrition and developmental milestones.

The findings revealed a consistent pattern across all three countries, wherein women receiving ANC in the first trimester were more likely to consume IFA tablets for at least 90 days compared to those who initiating ANC in the second or third trimester. Moreover, this study revealed that the number of ANC visits were associated with higher consumption of IFA tablets. This result aligns with prior studies of LMICs highlighting the importance of early ANC initiation for positive maternal health and nutrition outcomes [26,28,32–35]. The link between early and adequate ANC initiation and IFA consumption can be attributed to the provision of health education and counseling during these early visits, promoting awareness and adherence to micronutrient supplementation guidelines. This highlights the need for comprehensive and targeted interventions, like community health education, improved access to healthcare, early counseling, to ensure adequate care and support for expectant mothers throughout pregnancy.

This study revealed that while ANC timing did not directly influence early breastfeeding initiation across all countries, ANC frequency played an important role. Consistent with other studies, women with four or more ANC visits were more likely to initiate breastfeeding early [36–38], highlighting the multifaceted nature of ANC’s impact on maternal health behaviours. This may be because health professionals provide antenatal guidance and counseling on breastfeeding [39]. This is supported by our findings that the likelihood of delayed breastfeeding initiation was significantly lower among mothers who received breastfeeding advice during ANC visits in all countries. This finding underscores the importance of ANC as a platform for educating expectant mothers about optimal breastfeeding practices and maternal nutrition. The inverse association between ANC frequency and delayed initiation implies that more frequent ANC visits expose mothers to greater information and support, thereby promoting timely breastfeeding initiation.

Contrary to other studies [40,41], the study did not establish a statistically significant connection between ANC timing and frequency and exclusive breastfeeding practices. This underscores that while ANC may have a strong impact on initial breastfeeding practices, the factors driving EBF decisions might be more complex and influenced by a range of social, cultural, and environmental factors [42,43]. The impact of ANC timing and frequency might be overshadowed by these cultural and social factors in some settings. Variations in ANC services, healthcare systems, and breastfeeding promotional efforts across regions could explain why ANC visits might not predict EBF outcomes in some contexts [21,43–45]. However, the non-significant relationship between ANC and EBF does not necessarily invalidate the importance of ANC; rather it emphasizes the need for a more holistic approach to understanding breastfeeding behaviours. The finding that receiving breastfeeding advice during ANC visits was linked to higher odds of EBF among Indian mothers emphasizes the potential for targeted counseling interventions to promote EBF, particularly in settings where the early introduction of complementary foods is common [21,44,46].

Findings from this study have notable implications for policy and program. Strengthening ANC services, particularly by emphasizing early initiation and comprehensive nutrition education, is crucial for improving maternal micronutrient intake and breastfeeding practices. Healthcare providers play a vital role in promoting positive maternal behaviours and should be adequately trained to provide effective counseling during ANC. The study suggests that the effectiveness of ANC visits and breastfeeding advice remains effective during pregnancy and immediately after childbirth, not for a long time after delivery. Evidence shows that infant feeding counseling during postnatal care has been associated with a longer duration of EBF [47]. Therefore, emphasis should be given to both antenatal and postnatal care for providing counseling on maternal and child health and influencing mothers’ EBF practices and continuity.

The findings underline the potential of ANC to enhance maternal behaviours, especially in LMICs with suboptimal maternal and child health indicators. Enhancing ANC services, particularly in terms of early initiation and frequent visits, can lead to improvements in maternal micronutrient intakes and breastfeeding practices. Moreover, integrating targeted counseling on maternal nutrition and breastfeeding during ANC visits can yield substantial benefits for both mothers and infants. Despite the significant insights provided by this study, several limitations must be acknowledged. First, the study relies on cross-sectional data, limiting our ability to establish causal relationships. Second, the data are self-reported, introducing the possibility of recall bias and/or social desirability bias. Maternal recall, as a subjective measure, may introduce recall bias affecting the accuracy of reported data on ANC utilization, IFA intake, and breastfeeding practices. A recent study found moderate individual-level validity and low population bias in maternal recall of any IFA receipt during ANC, but substantial bias, including overreporting, in recalling the number of IFA tablets received, leading to inaccurate coverage estimates [48]. The validity of maternal recall during DHS is important as inaccurate recall can potentially impact program effectiveness, policy decisions, resource allocation, and research findings. Future research and policy decisions should consider methodological improvements, including validation studies, enhanced questionnaire design, objective measures, and longitudinal data collection to ensure the accuracy of maternal recall. Third, we acknowledge the omission of sample weighting in our analysis, underscoring its potential impact on the generalizability of our findings, and advocate for future research to explore the implications of weighting on observed associations. Finally, the study focused on a limited set of variables, potentially omitting other factors that may affect maternal health behaviours. Longitudinal studies are warranted to investigate the long-term influence of ANC engagement on maternal and child health outcomes.

## Conclusion

This secondary analysis of Demographic and Health Surveys data from Bangladesh, India and Pakistan provides valuable insights into the connection between ANC utilization and maternal micronutrient consumption and breastfeeding practices. The findings highlight the significance of both ANC timing and frequency, as well as the provision of breastfeeding advice, while also emphasizing the need for tailored interventions based on country-specific disparities, healthcare infrastructure, and cultural norms. As South Asia continues to grapple with the burden of malnutrition and micronutrient deficiencies, optimizing ANC services can contribute to breaking the cycle of intergenerational malnutrition and improving the health of mothers and their children. This study advocates for public policy interventions that promote early ANC initiation with an adequate number of visits, ensure sufficient distribution of micronutrient supplements, and provide health education during ANC visits to improve maternal and child health outcomes in these regions and similar settings.

## Supporting information

S1 Table Socio-demographic, timing and frequency of antenatal care, and micronutrient consumption and breastfeeding performance of the study participants: Demographic and Health Surveys Bangladesh, India, and Pakistan. (DOCX)
