# Supplementary material for: Do timing and frequency of antenatal care make a difference in maternal micronutrient intake and breastfeeding practices? Insights from a multi-country study in South Asia
Source: PLOS Glob Public Health. 2024 Mar 4;4(3):e0002993. doi: 10.1371/journal.pgph.0002993 (PMC10911624; doi:10.1371/journal.pgph.0002993)
Supplement: S1 Table — (DOCX) [file pgph.0002993.s001.docx]

**S1 Table**: Socio-demographic, timing and frequency of antenatal care, and micronutrient consumption and breastfeeding performance of the study participants: Demographic and Health Surveys Bangladesh, India, and Pakistan

|  | Bangladesh | | | India | | | Pakistan | | |
| --- | --- | --- | --- | --- | --- | --- | --- | --- | --- |
| Characteristic | Consumption of IFA (%) | EIB (%) | EBF (%) | Consumption of IFA (%) | EIB (%) | EBF (%) | Consumption of IFA (%) | EIB (%) | EBF (%) |
| Age, years  15-19  20-24  25+  *p-*Value | 54.5  67.3  66.8  0.009 | 51.9  56.0  56.9  0.47 | 54.5  53.2  59.2  0.71 | 44.2  48.9  52.9  <0.001 | 47.3  44.9  46.8  <0.001 | 29.4  19.5  16.8  <0.001 | 39.7  50.3  57.4  0.012 | 27.0  21.0  23.2  0.44 | 56.0  47.8  45.2  0.44 |
| Education  No education  Primary education  Secondary Education  Higher Education  *p-*Value | 33.3  50.3  64.9  75.8  <0.001 | 48.7  62.1  55.3  51.9  0.15 | 66.7  64.3  50.9  59.0  0.36 | 36.6  42.9  54.5  67.4  <0.001 | 41.6  46.5  49.4  43.1  <0.001 | 19.4  19.1  19.2  18.4  0.54 | 40.8  46.7  50.8  75.8  <0.001 | 23.6  23.4  22.9  21.8  0.97 | 61.2  57.6  38.2  24.6  <0.001 |
| Area of residence  Urban  Rural  *p-*Value | 64.9  63.7  0.72 | 53.6  57.3  0.26 | 57.9  54.3  0.58 | 59.9  47.8  <0.001 | 45.0  46.8  <0.001 | 16.5  20.0  <0.001 | 60.5  45.2  <0.001 | 24.6  21.6  0.26 | 40.8  54.1  0.03 |
| Wealth index category  Poor  Middle  Rich  *p-*Value | 59.3  63.3  66.3  0.31 | 64.9  53.4  53.1  0.04 | 50.0  54.2  59.0  0.55 | 39.6  52.5  62.6  <0.001 | 46.7  49.0  44.3  <0.001 | 20.9  18.3  17.4  <0.001 | 44.8  41.3  64.2  <0.001 | 25.1  22.6  21.1  0.43 | 63.2  43.1  32.7  <0.001 |
| Pregnancy intendedness  Intended  Unintended  *p-*Value | 64.3  64.0  0.96 | 54.9  58.7  0.40 | 56.3  55.9  0.96 | 52.1  40.6  <0.001 | 46.6  41.2  <0.001 | 19.1  19.5  0.63 | 53.8  47.7  0.43 | 22.8  26.5  0.49 | 47.6  58.3  0.47 |
| Frequency of watching TV  Not at all  Irregularly  Regularly  *p-*Value | 70.7  70.0  63.7  0.57 | 61.7  50.0  53.3  0.60 | 58.8  50.0  56.3  0.93 | 37.1  45.2  58.5  <0.001 | 42.6  47.7  47.5  <0.001 | 21.1  19.0  18.1  <0.001 | 44.6  52.5  58.9  0.003 | 22.6  29.0  22.2  0.30 | 61.9  37.8  36.9  <0.001 |
| Number of ANC visits  <4 visits  ≥4 visits  *p-*Value | 49.2  71.5  <0.001 | 56.2  55.0  0.71 | 56.1  54.4  0.96 | 37.9  60.7  <0.001 | 42.3  49.9  <0.001 | 19.6  18.6  0.02 | 33.7  61.5  <0.001 | 25.0  21.7  0.24 | 55.9  43.3  0.05 |
| Timing of first ANC  First Trimester  Second Trimester  Third Trimester  *p-*Value | 72.9  60.3  44.8  <0.001 | 52.7  56.7  59.3  0.31 | 63.5  47.9  61.3  .0.08 | 56.5  43.4  49.6  <0.001 | 47.8  45.1  47.6  <0.001 | 18.7  18.7  22.1  .005 | 58.4  47.0  3.8  <0.001 | 20.6  21.7  19.1  0.91 | 44.2  56.3  41.7  .0.31 |
| Received Breastfeeding Counselling  No  Yes  *p-*Value |  | 51.9  48.9  0.57 | 76.5  54.7  0.09 |  | 40.3  50.9  <0.001 | 17.8  19.4  0.02 |  | 16.7  24.7  0.005 | 55.3  41.5  0.03 |

*Note: p*-values refer to differences between groups.

*ANC* Antenatal Care; *EIB* Early initiation of breastfeeding; *EBF* Exclusive breastfeeding
